# Supplementary material for: Multi-Omics Profiling to Assess Signaling Changes upon VHL Restoration and Identify Putative VHL Substrates in Clear Cell Renal Cell Carcinoma Cell Lines
Source: Cells. 2022 Jan 29;11(3):472. doi: 10.3390/cells11030472 (PMC8833913; doi:10.3390/cells11030472)
Supplement: Supplementary file 1 [file cells-11-00472-s001.zip › Supplementary Figures.pdf]

Supplementary Figures

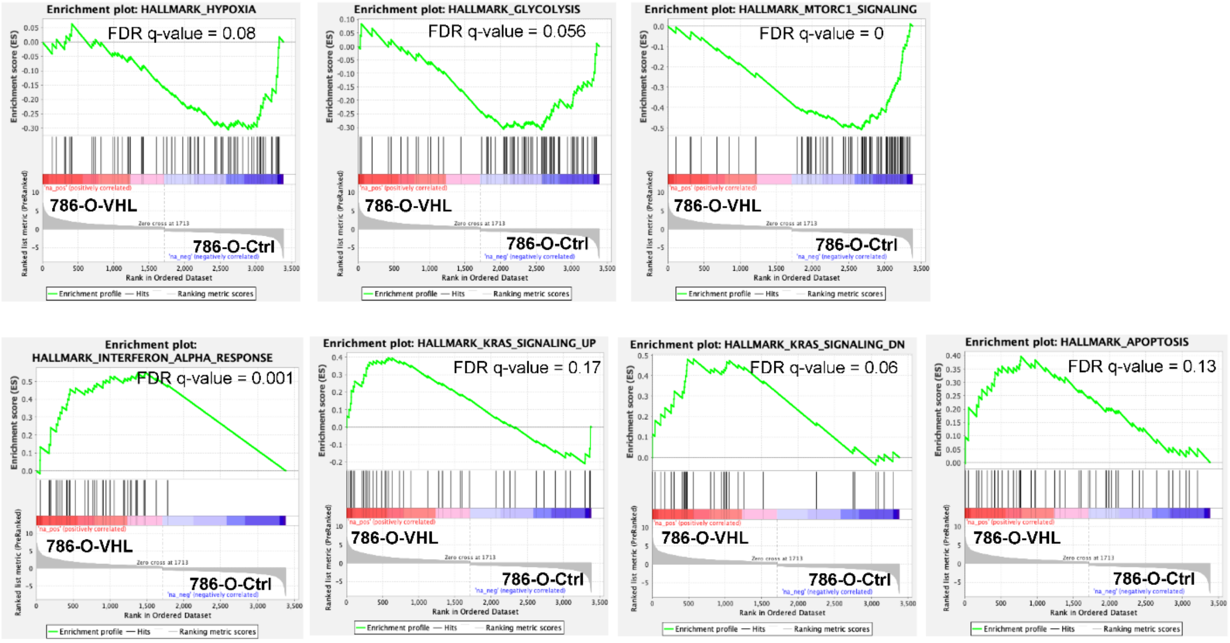

Supplementary Figure S1. Enrichment plots for GSEA hallmarks pathways significantly enriched to 786-O-Ctrl or 786-O-VHL based on RNA-seq data. Hypoxia, Glycolysis, and mTORC1 signaling pathways enriched in 786-O-Ctrl (FDR < 0.25). KRAS Signaling up and down, Apoptosis, and Interferon alpha response pathways enriched in 786-O-VHL (FDR < 0.25).

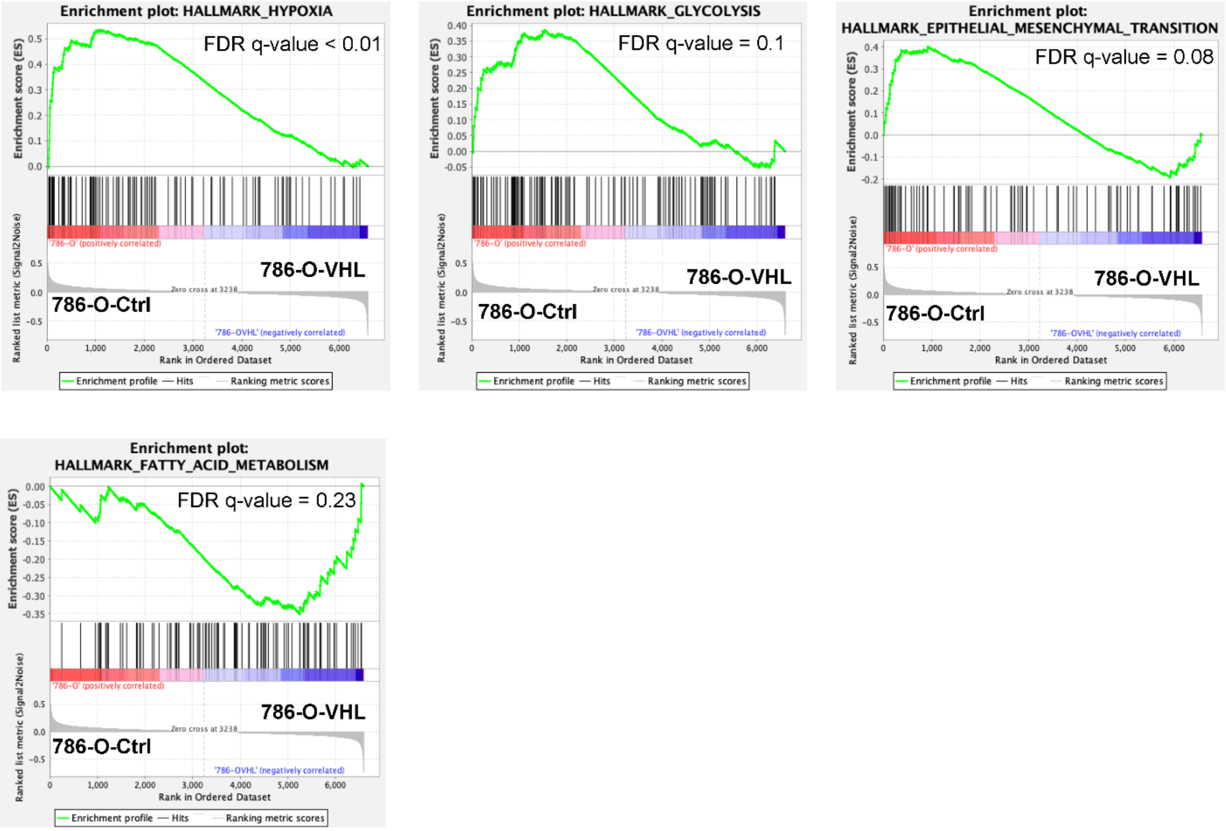

Supplementary Figure S2. Enrichment plots for GSEA hallmarks pathways significantly enriched to 786-O-Ctrl or 786-O-VHL based on proteomics data. Hypoxia, Glycolysis, and Epithelial mesenchymal transition pathways enriched in 786-O-Ctrl (FDR < 0.25). Fatty acid metabolism pathway enriched in 786-O-VHL (FDR < 0.25).

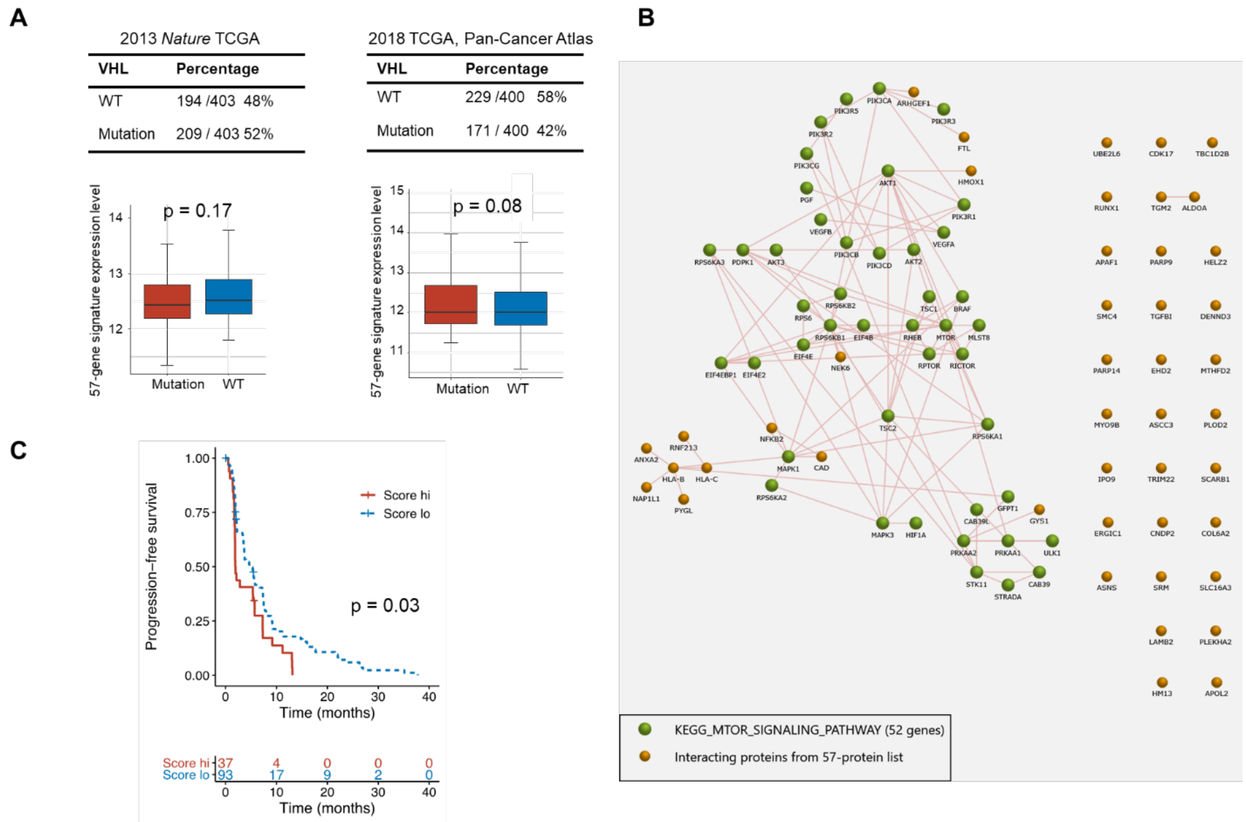

Supplementary Figure S3. Relationship of the 57-gene list and mTOR signaling. (A) The transcript level of the 57-gene signature in VHL-mutation and VHL-WT tumors of the ccRCC TCGA cohort (both 2013 and 2018), with clinical sample data downloaded from cBioportal. P value by t test. **(B)** Protein-protein interaction network constructed by FunRich to incorporate the 57 proteins on our list and the 52 proteins in the KEGG mTOR signaling pathway. **(C)** Kaplan-Meier analysis of progression-free survival for patients treated with mTOR inhibitor everolimus as the control arm of the clinical trial CheckMate 025, with the patients stratified based on the mRNA expression score of the 57-gene signature. P value calculated from Cox regression analysis.

A

Random forest method (21 genes)

Gene signature	Random Forest Importance
LAMB2	3.34
ANXA2	3.33
FNDC3B	3.22
ERGIC1	3.18
ARHGEF1	3.17
HM13	3.07
NEK6	2.96
TBC1D2	2.88
PARP14	2.85
COL6A2	2.78
AMPD2	2.77
PPIP5K2	2.72
APAF1	2.69
ASCC3	2.62
NAP1L1	2.40
SLC16A3	2.31
IPO9	2.21
NFKB2	2.17
GYS1	2.16
PYGL	2.15
TGFBI	2.08

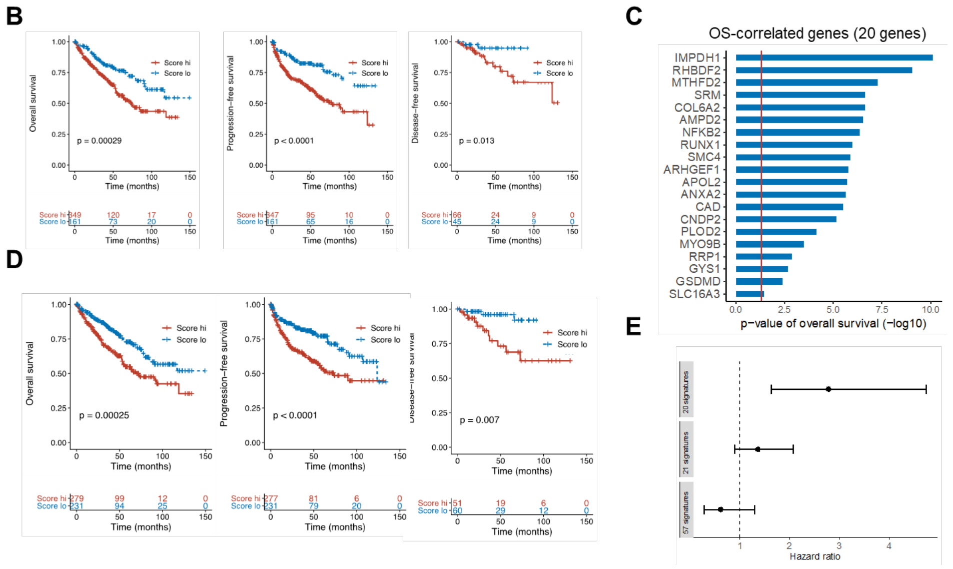

Supplementary Figure S4. Trimming down the 57-gene list for stronger prognosis. (A) Trimmed list of 21 genes through ranking the random forest importance values. **(B)** Kaplan-Meier analysis for overall survival, progression-free survival and disease-free survival for patients of the TCGA ccRCC cohort based on the 21-gene signature at the mRNA level. P values calculated by Cox regression analysis. **(C)** Trimmed list of 20 genes through ranking of significant correlation with patient survival using lasso feature selection, based on TCGA ccRCC transcriptome data. Genes ranked by P value with the threshold 0.05. **(D)** Kaplan-Meier analysis for overall survival, progression-free survival and disease-free survival for patients of the TCGA ccRCC cohort based on the 20-gene signature at the mRNA level. P values calculated from Cox regression analysis. **(E)** Hazard ratios of the 57-gene, 21-gene and 20-gene signatures, assessed in the ccRCC TCGA patient cohorts. Cox proportional hazards models are used to evaluate the association between gene signatures and survival.
